# Is Protein Folding Sub-Diffusive?

**DOI:** 10.1371/journal.pcbi.1000921

**Published:** 2010-09-16

**Authors:** Sergei V. Krivov

**Affiliations:** Institute of Molecular and Cellular Biology, University of Leeds, Leeds, United Kingdom; State University of New York at Stony Brook, United States of America

## Abstract

Protein folding dynamics is often described as diffusion on a free energy surface considered as a function of one or few reaction coordinates. However, a growing number of experiments and models show that, when projected onto a reaction coordinate, protein dynamics is sub-diffusive. This raises the question as to whether the conventionally used diffusive description of the dynamics is adequate. Here, we numerically construct the optimum reaction coordinate for a long equilibrium folding trajectory of a Go model of a 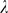-repressor protein. The trajectory projected onto this coordinate exhibits diffusive dynamics, while the dynamics of the same trajectory projected onto a sub-optimal reaction coordinate is sub-diffusive. We show that the higher the (cut-based) free energy profile for the putative reaction coordinate, the more diffusive the dynamics become when projected on this coordinate. The results suggest that whether the projected dynamics is diffusive or sub-diffusive depends on the chosen reaction coordinate. Protein folding can be described as diffusion on the free energy surface as function of the optimum reaction coordinate. And conversely, the conventional reaction coordinates, even though they might be based on physical intuition, are often sub-optimal and, hence, show sub-diffusive dynamics.

## Author Summary

To understand dynamics of complex systems with many degrees of freedom, one often projects it onto one or several collective variables. Protein folding, the complex, concerted motion of a protein chain towards a unique three-dimensional structure, is one example of where such reduction of complexity is useful. It is usually assumed that the projected dynamics is diffusive. However, many experiments and simulations have shown that the projected dynamics is sub-diffusive, i.e., the mean square displacement grows slower than linear with time. It means that the dynamics has a memory; that the free energy surface together with diffusion coefficient do not properly define the dynamics; and that such projections cannot be used to accurately describe dynamics. Here, we show that if one carefully constructs the reaction coordinate by optimizing (maximizing) its free energy profile, one can use a simple (memory-less) diffusive description. Loosely speaking, when the complex dynamics is projected onto a simple coordinate, all the complexity of the original dynamics goes into the memory of the projected dynamics. If the dynamics is projected onto the (complex) optimum reaction coordinate, all the complexity of the original dynamics is in the reaction coordinate, and the projected dynamics is simple.

## Introduction

A free energy surface (FES) projected onto one or a small number of coordinates is often used to describe the equilibrium and kinetic properties of complex systems with a very large number (100 to 1,000 or more) of degrees of freedom. Studies of protein folding are an important case where this type of projected surface has been introduced and coordinates such as the number of native contacts and radius of gyration have been used [1]–[3]. Protein folding then is described as diffusion on the projected free energy surface. Diffusive dynamics is characterized by means square displacement linearly growing with time, 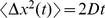, where D is the diffusion coefficient. For a single reaction coordinate diffusive dynamics is completely specified by the free energy profile (FEP), i.e. the free energy as a function of the coordinate and coordinate-dependent diffusion coefficient, which conveniently can be computed from conventional and cut based free energy profiles [4]. Construction of a “good” reaction coordinate (i.e. the one that preserves systems dynamics) is challenging. In many cases, the standard progress variables (e.g. number of native contacts, radius of gyration, root mean square distance from the native structure) are not good reaction coordinates, because they do not preserve the barriers on the FES and thus may mask the inherent complexity of the latter [5]. A number of methods to construct good reaction coordinates have been suggested [4], [6]–[9].

Employing the Mori-Zwanzig formalism [10], [11] one can derive generalized Langevin equations, which describe system dynamics projected on the reaction coordinates. The generalized Langevin equation contains a memory kernel, which leads to non-Markovian dynamics and subdiffusion. Subdiffusion is characterized by the mean square displacement growing slower than that for diffusion, 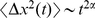 with exponent 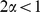. To completely specify dynamics in this case one has to compute the memory kernel, which is not trivial, since it requires the solution of a multidimensional partial differential equation [12]. Long-term memory in correlation functions and anomalous diffusion in proteins was observed experimentally and theoretically [13]–[23]. This raises the question whether the folding dynamics of proteins can be described as simple diffusion on the projected free energy surface, as is often done, or if one has to use more sophisticated descriptions, e.g. generalized Langevin equations [24], [25], fractional Fokker-Plank equations [26] or multiscale state space networks [19]. Here we show that if the reaction coordinate is properly optimized, then the dynamics projected onto this coordinate is diffusive, while the same dynamics projected onto a sub-optimal coordinate is sub-diffusive.

## Results/Discussion

The equilibrium folding dynamics of the 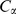 Go model [27] of the N-terminal domain of phage 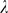-repressor protein is analyzed [28]. Structure-based Go models containing attractive native interactions and repulsive nonnative interactions correspond to perfectly funneled energy landscapes with energetic frustration completely absent [3], [29]. A trajectory of 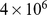 frames (saved with 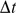 =  7.5 ps) was obtained by simulating with Langevin molecular dynamics at T = 323 K and contains about 100 folding-unfolding events. The saving interval of 7.5 ps is used below as the unit of time. Note that the timescales in the simulation do not correspond directly to the timescales of the folding dynamics of the real protein because the coarse-grained model of the protein without explicit representation of the solvent is employed. Relation between the folding timescales of coarse-grained models of proteins and that of real proteins is discussed in [30]. The protein has complex FES with five basins: denatured, native, native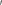, intermediate and intermediate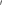 and two symmetrical folding pathways [28].

Optimum one-dimensional reaction coordinates are constructed by numerically optimizing the mean first passage time to the native basin for a sufficiently broadly chosen functional form of a reaction coordinate (see Methods). Two different functional forms of reaction coordinates are considered. For each coordinate we show the cut based free energy profile (FEP) 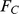 together with the exponent 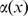; the latter is used to distinguish between diffusive and sub-diffusive dynamics (see Methods). The coordinate dependent exponent 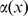 describes how the mean absolute displacement grows with time, 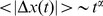 and can be determined from the distance between 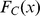 computed at two different sampling intervals 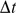 (see Methods); the smaller is the distance, the higher is the exponent 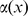. 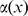 is equal to 1/2 for diffusive and is less than 1/2 for sub-diffusive dynamics. Each coordinate is transformed to the natural coordinate (see Methods), so that the diffusion coefficient is constant and is equal to one and diffusive dynamics is completely specified by the FEP 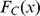.

**Figure 1:**
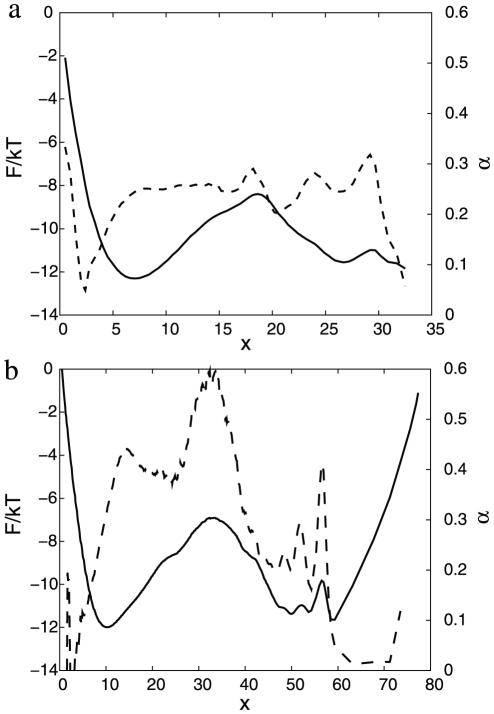
Optimization of 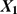 reaction coordinate. 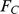
 (solid line) and 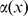 (dashed line) for 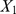 as a reaction coordinate; (a) 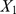 initialized to NNC, (b) optimized 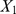. Reaction coordinates are transformed to the natural reaction coordinate.

**Figure 2:**
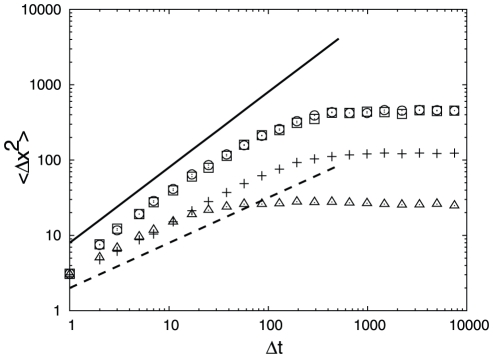
MSD for pieces of the trajectory starting from the corresponding transition states. Pluses are for unoptimized 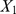, squares are for optimized 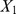, triangles are for unoptimized 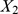, circles are for optimized 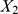. The solid line shows diffusive (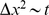) and the dashed line sub-diffusive (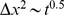) MSD to guide the eye.

The first coordinate (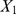) generalizes the number of native contacts coordinate NNC as: 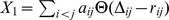, where 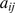 is either 1 or −1, 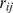 is the distance between atoms 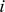 and 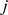 and 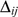 is the distance threshold, when contact between the atoms is considered to be formed; 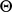 is the Heaviside step function, whose value is zero for a negative argument and one for a positive argument. Figure 1a shows 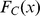 and 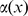 for the (sub-optimal) reaction coordinate, just initialized to the NNC, i.e., 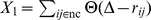 with sum over pairs of atoms (ij) in the set of native contacts. The value of 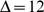 gives the highest barrier for the transition state for the simple variants of NNC, where the distance threshold is the same for all the native contacts. The relatively large value (inter-atom distances between 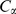 atoms in the native contacts are within 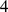 to 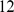 Å) may be explained by the fact that the optimal reaction coordinate should better distinguish between the denatured and native basins (rather than indicate a formed native contact), which happens around the transition state and sufficiently far from the native structure. On the FEP one can notice three basins: denatured 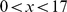, native 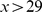; the third basin 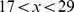 consists of a number of overlapping free energy basins. The exponent 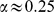 shows that the dynamics is sub-diffusive. To confirm this Figure 2 shows the mean square displacement (MSD) as a function of time (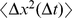) averaged over pieces of the trajectory that start from the transition state (TS) (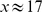). The MSD grows approximately as 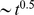 (the mean absolute displacement as 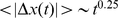), indicating sub-diffusive dynamics. The number of folding events computed with Kramer's equation (Eq. 4) is 1200, i.e. an order of magnitude more than the actual number of 100 events. It means that the reaction coordinate is “bad” and the computed folding free energy barrier is lower than the correct one. Limited structural information can be exploited by making the distance threshold proportional to the native distance 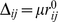 for each native contact (ij), so that 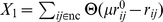. However, it does not improve the reaction coordinate since the highest barrier for the transition state, obtained at 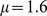 (see Figure S1 in Text S1), is similar to that obtained with the constant threshold (Figure 1a).

**Figure 3:**
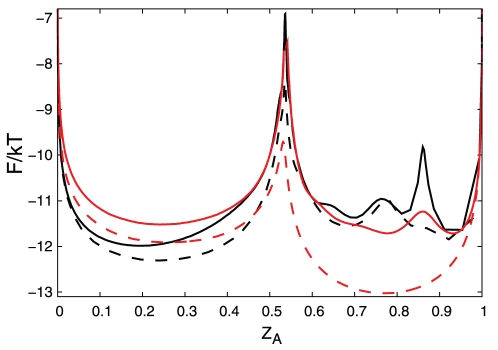
The reaction coordinates comparison. Black and red lines show the free energy profiles along the 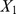 and 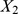 coordinates, respectively. Solid and dashed lines show optimized and non-optimized coordinates, respectively.

Figure 1b shows 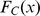 and 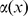 for the optimized reaction coordinate 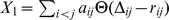. The FEP is more informative now: one can distinguish the three basins, that were overlapping on Figure 1a. The free energy of the transition state (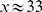) of the optimized reaction coordinate is higher than that for the sub-optimal one (Figure 1a). The relative position of the transition state for the optimum coordinate is shifted to the left compared to the NNC coordinate which may give a misleading impression that the transition states occupy different regions of the configuration space. The optimum and NNC reaction coordinates have different coordinate dependent diffusion coefficients. When the coordinates are transformed to the natural coordinate with diffusion coefficient equal to unity the same regions of the configuration space may occupy different positions. Figure 3 shows FEPs along the 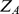 reaction coordinate, which is invariant to coordinate transformation, and can be used to compare different coordinates. 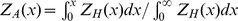 measures the relative partition function of the coordinate segment between 0 and x. The transition states on Figure 1 correspond to those on Figure 3, since the cut free energy profiles are invariant under coordinate transformation [4]. The transition states on Figure 3 are located at the same position, i.e., they occupy the same region of the configuration space. 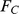 for the optimum coordinates are uniformly higher than that for the corresponding sub-optimum ones. 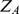 coordinate, however, is of limited use to correctly represent the dynamics since the diffusion coefficient is not constant, which leads to such artifacts as sharply peaked transition states.

The scaling exponent 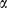 for the optimized reaction coordinate (Figure 1b) is no longer a constant. It is a bit higher than 0.5 at the TS region (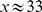) and a bit lower than 0.5 in the denatured state and at the second barrier (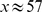), indicating diffusive dynamics. After the TS 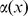 is around 0.25 indicating sub-diffusive dynamics. Values of 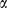 higher than 0.5 (superdiffsion) are an artifact due to over-fitting of the trajectory by the reaction coordinate. The estimated number of folding events for the optimized reaction coordinate is 168, which is quite close to the actual number. Figure 2 shows MSD for the pieces of the trajectory starting from the TS (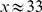). The MSD grows linearly with time, confirming diffusive dynamics. The reaction coordinate can be optimized in another region, e.g. by maximizing the mfpt to go from the TS (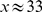) to the native structure (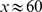). In that case dynamics in the region around the second barrier (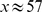) becomes diffusive, while that at the TS is back to sub-diffusive. Optimization of the reaction coordinate inside the native basin has increased the exponent 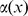 in the basin from 0 to 0.3, indicating that the dynamics in the basin is still sub-diffusive. This can be due to a relatively large value of the sampling interval (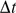) of 7.5 ps, at which MSD between two subsequent snapshots is close to an equilibrium value inside the native basin. Moreover, sub-diffusive dynamics inside the basins have relatively small influence on folding dynamics, which is determined mainly by diffusive dynamics at the transitions state regions. The Text S1 shows an all-atom structure based model of the lambda repressor protein where the optimum reaction coordinate is constructed so that the dynamics is diffusive for the whole coordinate, not just around the transition state.

**Figure 4:**
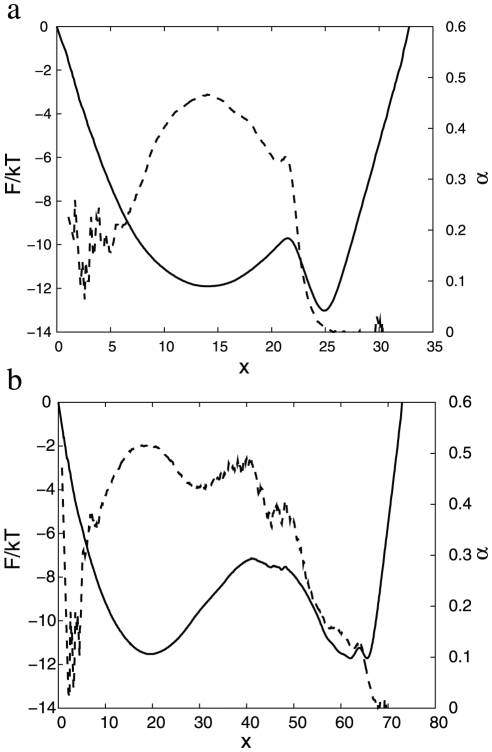
Optimization of 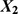 reaction coordinate. 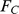
 (solid line) and 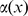 (dashed line) for 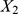 as a reaction coordinate; (a) 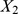 initialized to 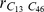, (b) optimized 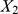. Reaction coordinates are transformed to the natural reaction coordinate.

The second coordinate is a linear combination of all interatom distances 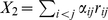, where 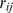 is the distance between atoms 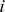 and 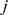. It was initialized to be the distance between atoms 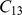 and 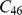 (Figure 4a). The end to end distance (the distance between 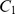 and 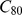 atoms), often employed in single molecule experiments, does not separate the denatured and native basins; the free energy profile along the distance is barrier-less. Figures 4 and 2 show 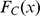, 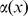 and MSD for 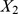 and lead to the similar conclusions. For the sub-optimal 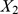 dynamics is sub-diffusive at the TS (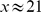), with the exponent 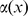 steeply decreasing to zero just after the TS. The exponent 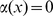 means that the MSD has reached the equilibrium value (at this time scale and in this region of the reaction coordinate). The estimated number of folding events is about 8700. The optimized reaction coordinate (panel b) has a higher folding barrier and shows that the dynamics is diffusive at the TS (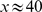) and estimates the number of folding events as 154.

**Figure 5:**
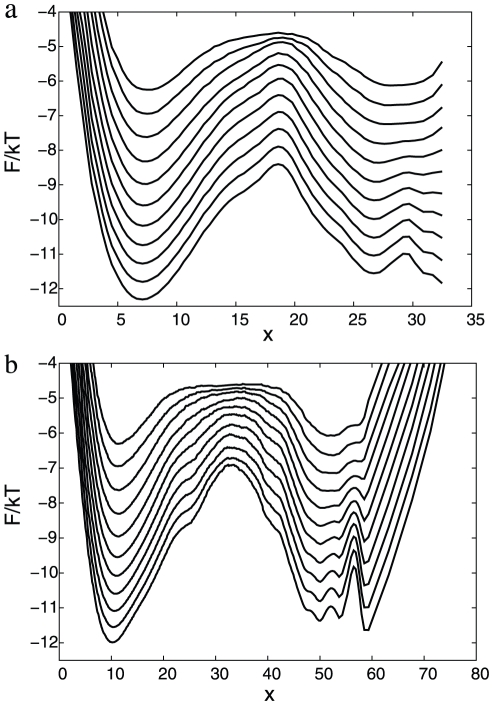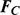 computed at different sampling intervals for 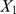 as a reaction coordinate. The sampling intervals are 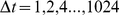; (a) the NNC reaction coordinate, (b) the optimum reaction coordinate. Higher free energy barrier for the optimum reaction coordinate implies lesser space between the profiles and larger 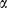 compared to the NNC.

**Figure 6:**
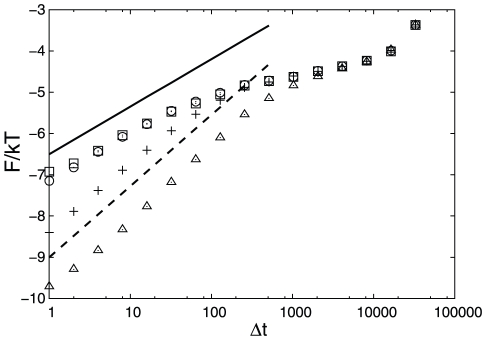
Scaling of 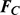 at the transition state with the sampling interval. The sampling intervals are 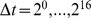. 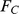 of the TS are shown by symbols; notation as in Figure 2. The solid line shows the diffusive slope (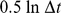) and the dashed line shows the sub-diffusive slope (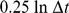) to guide the eye.

Figure 5 shows 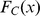 for different values of the sampling interval 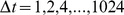 for the optimal and sub-optimal coordinates 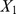. The constant distance between the profiles at fixed 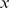 and different (small) 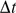 means that 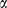 is independent of 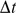 and that 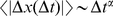 (see also Figure 6).

The partition function of the cut based free energy profiles 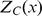 (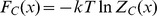) at point 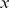 is defined as the number of transitions through the point [4] (see Methods). For the sufficiently large sampling intervals 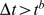, when the system “flies” ballistically over the TS barrier, i.e. no recrossing events are detected, the 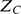 at the TS is equal to the total number of folding events (100 here). This value denoted as 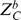 (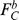) is, evidently, the same for the optimal and sub-optimal coordinates. The optimum reaction coordinate has higher 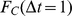 at the TS compared to the sub-optimal coordinate. Hence, 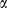 (at the TS) estimated as (see Methods)
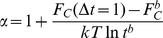
(1)is higher for the optimum reaction coordinate than it is for the sub-optimal one. In other words, an inadequacy of a sub-optimal reaction coordinate (low 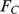) which leads to faster kinetics is corrected by making the dynamics sub-diffusive (slower).

We assume here that 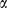 is roughly a constant for the sampling intervals between 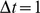 and 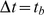 (i.e., 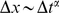), which is validated by Figure 5. However the assumption evidently breaks down for the very small time scales, when the system follows Newtons equations of motion with 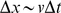 meaning 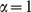. Thus, the sampling interval 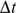 should be chosen sufficiently large so that the dynamics is in the (sub)diffusive regime.

Figure 6 shows 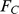 computed at the TS as a function of 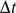 for different reaction coordinates. Initially, 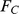 curves have a constant slope, which is close to diffusive for the optimized reaction coordinates and to sub-diffusive for the sub-optimal reaction coordinates. The slope changes when 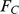 approaches the limiting value of 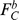. The latter is not strictly constant, though its dependence on 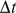 is rather weak. As 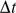 increases further (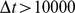, the mean life time in the basins), the probability of the system to visit another basin undetected (between successive sampling events) increases as well and 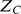 (the number of detected transitions) decreases. 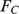 for different reaction coordinates fall on the same curve at sufficiently large 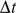, i.e. 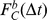 are the same when local differences between the coordinates become negligible.

However, Figure 6 shows that the ballistic time (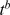) for different reaction coordinates is slightly different, while in deriving Eq. 1 it was assumed to be constant. To take this into account we proceed as follows. The curve 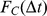 (Figure 6) is approximated by two straight lines as 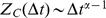 for 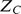 less than the limiting value of 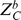 (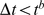) and constant 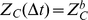 (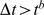), where 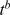 is the time when dynamics becomes ballistic. Define 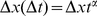, and 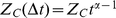; where 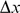 and 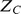 denote, respectively, 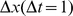 and 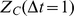. The diffusion coefficient is set to unity by transforming the reaction coordinate to the natural coordinate, which means that 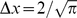. The time 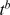 can be estimated as time when mean absolute displacement is about the barrier width (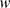), i.e. 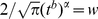. At this time 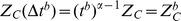. Eliminating 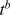 from the two equations, one finds
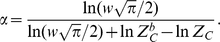
(2)Taking 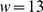 and 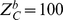, one obtains 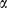 equal to 0.32, 0.39 and 0.49 for 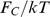 equal to −9.72, −8.34 and −7.13, respectively, in reasonable agreement with Figure 6. The ballistic times are 1924, 487 and 144, respectively. From Eq. 2 it follows that the higher is the free energy barrier the higher is the exponent 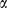 and the closer is the dynamics to the diffusive one.

The two optimized reaction coordinates, while having very different functional forms, show very similar behavior (at the TS regions), e.g. the width and the height of the TS barrier is the same (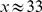 on Figure 1b and 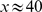 Figure 4b), the MSDs are identical (Figure 2) as well as 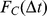 dependencies (Figure 6). This, likely, indicates that the two coordinates have converged to and closely approximate the true reaction coordinate (at the TS region). The residual difference between the estimated and the actual numbers of folding events which is due to limited statistics and insufficient flexibility of the chosen functional forms, is relatively small so it does not affect the results. The fact that the diffusive character of dynamics is determined by the height of free energy barrier 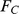, rather than the chosen functional form of the coordinate indicates the robustness of the approach. It also means that the method of constructing the optimum reaction coordinate by optimizing its FEP (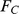) [4], [6] has an advantage over the other approaches [7]–[9], in that it guarantees that the optimum reaction coordinate has dynamics closest to diffusive. Distribution of folding times is single exponential and identical for all four coordinates because folding events can be detected with high likelihood by any sufficiently good order parameter.

The analysis suggests that the higher is the free energy profile the closer is dynamics to diffusive. Evidently, the most optimal reaction coordinate is the one which has its free energy highest for every value of reaction coordinate. Consider invariant parametrization of reaction coordinate, namely the partition function of the configuration space from the initial value to the position x 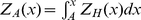. The optimum reaction coordinate is the one that attains 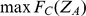 or 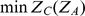 for any 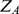, assuming that 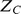 for different values of 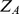 can be varied independently. This defines the optimum reaction coordinate introduced in [4], which has the largest mean first passage time. Conversely, diffusive dynamics on the constructed reaction coordinate can serve as an indication of optimality of the reaction coordinate.

To illustrate that the results presented are robust with respect to particular choice of the protein or the interaction potential, a protein with different secondary structure content (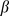-sheet) and an all-atom structure based model of the lambda repressor protein are analyzed in Text S1. The analysis confirms that the dynamics is sub-diffusive when projected onto a sub-optimum reaction coordinate and diffusive, when projected onto the optimum reaction coordinate.

Low free energy barrier *per se* does not mean that the dynamics is sub-diffusive, for example, a freely diffusing particle has flat free energy profile. Dynamics should be sub-diffusive, when the reaction coordinate is sub-optimal, i.e., the free energy barrier along the coordinate is much lower than the correct one. The latter is defined either as the highest barrier attained by the optimum reaction coordinates, or as a solution of the multidimensional minimum cut problem (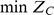), which locates the transition state [4].

The analysis above just considers the dynamics around the transition state, i.e., at the top of the free energy barrier. The conclusion that the higher the free energy profile the closer the dynamics to diffusive is likely to be valid in general, e.g., for the barrier-less folding proteins. The quantitative analysis exploits the fact that at the very large sampling intervals, when the system flies ballistically over the barrier, the two free energy profiles for optimal and sub-optimal reaction coordinates are very similar, because the two coordinates distinguish equally well between the basins. It can be extended to the following general qualitative argument. The two sufficiently good reaction coordinates likely differ significantly only at relatively small spatial scales with the large scale description of the dynamics being very similar. As the sampling interval 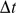 increases, the characteristic change of the reaction coordinates during the sampling interval (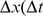)) increases as well. When (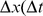)) is comparable to the large scale, so that the relative difference between the coordinate is negligible, the description of the dynamics by the two coordinates is similar and results in similar free energy profiles. Since the distance between the higher profile and the joint profile at large sampling intervals is smaller than that for the lower profile, the dynamics in former case is closer to diffusive compare to the later. It is assumed that 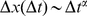 is valid for the whole range of 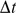 from the small sampling intervals, when the dynamics start to manifests itself as (sub)diffusive to the large sampling intervals, where the profiles for the different reaction coordinates become very similar. This equation connects the dynamics and the free energy profiles at these different time scales.

The model of the protein employed in the analysis is relatively simple, thus allows for extensive simulation with large number of folding-unfolding events. More realistic simulation of protein folding would include explicit representation of solvent configuration degrees of freedom. The dynamics projected on the optimum reaction coordinate constructed by considering only protein degrees of freedom might be sub-diffusive because neglected solvent degrees of freedom could be important.

The analysis suggests that without specifying the reaction coordinate, the question why the dynamics is sub-diffusive is rather ill-posed. It is more appropriate to ask: is it possible, for a given trajectory, to construct the optimum reaction coordinate, so that the projected dynamics is diffusive?

In conclusion, we have shown that dynamics projected onto a reaction coordinate can be diffusive or sub-diffusive depending on the coordinate employed for the projection. If one has a flexibility in choosing the reaction coordinate, e.g. when describing protein folding, dynamics can be made diffusive (or close to it) by optimizing the reaction coordinate (making 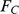 higher). When the coordinate describing the process is specified and can not be varied, for example, the donor-acceptor distance in the single molecule FRET or ET experiments [24], [31] or the mean square displacement in the neutron scattering experiments [13], the dynamics is likely to be sub-diffusive [13], [14], [31]. However, this does not necessarily mean that the dynamics *per se* is sub-diffusive. A properly chosen reaction coordinate (too complex to realize in experiment) may show that dynamics of transition between free energy basins is diffusive. A relatively small deficiency of the putative reaction coordinate (difference in 1 kT in free energy (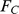) of the folding barrier) is sufficient to make the dynamics sub-diffusive. Hence, one should model protein dynamics as diffusion on a putative reaction coordinate [32], [33] with care, because, it is very likely that the coordinate is sub-optimal, unless it has been specifically constructed (optimized) [5], [28].

## Methods

### Free energy profiles

The conventional way to construct the FEP, given the projection of a trajectory onto a reaction coordinate (the time-series of the value of the reaction coordinate) 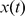, is to compute a histogram and estimate the partition function (probability density) as 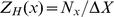, where 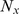 is the number of time-series points in bin 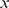 and 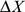 is the size of the bin. The free energy can then be found as 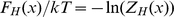. The partition function of the cut based free energy profile [4] at point 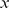 is defined as the number of transitions through that point, i.e. 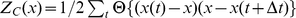, where 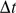 is the sampling interval and 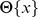 is the Heaviside step function; 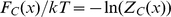. Assuming that the 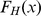 is approximately constant on the distance of the mean absolute displacement 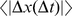, one can derive the following expression
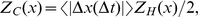
(3)where 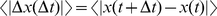 is the mean absolute displacement during sampling interval; for diffusive dynamics it gives 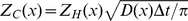.

A reaction coordinate (x) with a variable diffusion coefficient can be transformed to coordinate (y), called the natural coordinate [4], so that the diffusion coefficient is constant and equal to unity, by numerically integrating 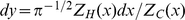; i.e. that 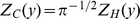.

Other approaches have been suggested to characterize diffusive dynamics by computing the free energy profile together with the coordinate dependent diffusion coefficient [32], [34], [35]. It is not clear, however, if they can be used to characterize the sub-diffusive regime.

### Reaction coordinate optimization

It is reasonable to assume that any “bad” choice of reaction coordinate, when different parts of the configuration space overlaps at projection onto this coordinate, will result in faster kinetics, i.e. in a smaller mean first passage time (mfpt). Clearly, the longest mfpt is obtained on the original FES or from a projection where no such overlapping occurs. Hence, we define the optimum reaction coordinate as the one that has the longest mfpt, which can be computed by Kramer's equation [4]

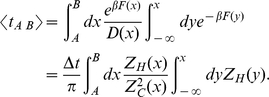
(4)


The optimum reaction coordinates are constructed by numerically optimizing the mfpt functional for a sufficiently broadly chosen functional form of reaction coordinate. Starting with the initial set of parameters, which are sufficient to distinguish between the two free energy basins, the coordinate is iteratively improved by changing parameters and accepting the change if mfpt is increased. For the first reaction coordinate 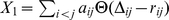 we pick a random pair of atoms 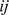, scan the whole parameter space for the pair (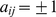 and 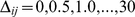) and select the one that gives the highest mfpt. For the second reaction coordinate 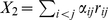 we pick a random pair 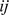, scan the whole parameter space for the pair (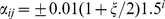 for 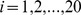 and 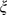 is a random number uniformly distributed between 0 and 1) and select the one that gives the highest mfpt. For the given values of parameters the mfpt is computed by first computing 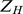 and 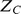 and then numerically integrating Eq. 4. Alternatively one may minimize the number of transitions, the quantity related to mfpt as 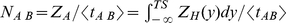, where 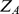 is the partition function of basin A and 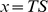 is the position of the transition state between basins A and B.

### Subdiffusion

For subdiffusion, the mean absolute displacement no longer scales as 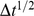, but rather as 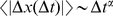. The exponent 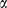 (possibly coordinate dependent), can be determined by comparing 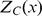 at two different sampling intervals (see Eq. 3). For a trajectory with fixed length and varying sampling interval 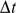 (when 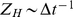) it is equal to
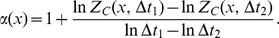
(5)Since 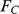 is invariant with respect to nonlinear coordinate transformation, the scaling exponent 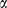 computed by Eq. 5 is also invariant, while 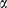 computed from 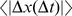 or 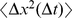 are not invariant and are computed here after the coordinate has been transformed to the natural reaction coordinate.

## Supporting Information

Text S1 Supporting information for “Is Protein Folding Sub-Diffusive?”.
(0.08 MB PDF)
