# Supplementary material for: Is Protein Folding Sub-Diffusive?
Source: PLoS Comput Biol. 2010 Sep 16;6(9):e1000921. doi: 10.1371/journal.pcbi.1000921 (PMC2940728; doi:10.1371/journal.pcbi.1000921)
Supplement: Text S1 — Supporting information for “Is Protein Folding Sub-Diffusive?”. (0.08 MB PDF) [file pcbi.1000921.s001.pdf]

Supporting Information for *Is Protein Folding Sub-Diffusive?*

Sergei V. Krivov,
Institute of Molecular and Cellular Biology, University of Leeds, Leeds, United Kingdom
E-mail: s.krivov@leeds.ac.uk

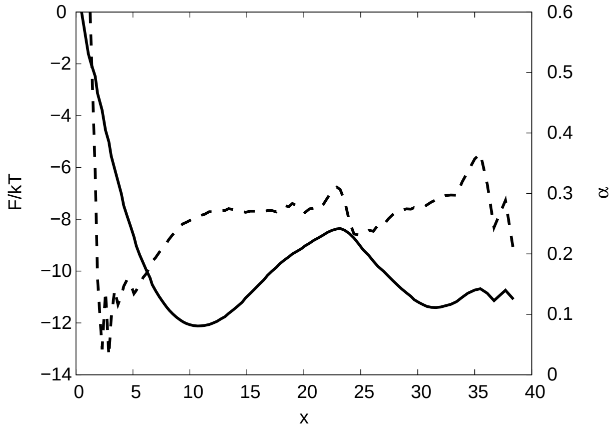

Figure 1. Optimization of X_1 reaction coordinate for λ-repressor. The number of native contacts reaction coordinate X_1 with the distance thresholds proportional to the native distance for each native contact $X_1 = \sum_{ij} \Theta(\mu r^0_{ij} - r_{ij})$; $\mu = 1.6$ gives the highest transition state barrier. $F_C(x)$ and $\alpha(x)$ are shown by solid and dashed lines, respectively.

To illustrate that the results presented are robust a protein with different secondary structure content (β-sheet) and a model of the lambda repressor protein with a more fine-grained (all atom) force field have been analyzed.

All-β protein

Equilibrium folding simulation of the (all beta) E2lim3 protein [1] was performed with the C_α structure based Go potential at equilibrium temperature of 300K. A trajectory of 4×10^5 frames (saved every 75 ps) was obtained by simulating with Langevin molecular dynamics.

Fig. 2a shows the FEP along the number of native contacts reaction coordinate, with the distance thresholds proportional to the native distance for each native contact $X_1 = \sum_{ij} \Theta(\mu r^0_{ij} - r_{ij})$; $\mu = 2$ gives the highest barrier at $x = 25$. The FEP has three basins: denatured $x < 15$, intermediate $18 < x < 24$, and native $26 < x$. Dynamics around the highest barrier $x = 25$ is diffusive, as is shown by $\alpha \approx 0.5$. One may conclude that this barrier is the transition state, and that the constructed reaction coordinate provides accurate, diffusive description of the folding dynamics at least around the "transition state" ($x = 25$). However, this reaction coordinate appears to be sub-optimal and the landscape picture is qualitatively wrong. The optimum reaction coordinate is constructed by optimizing the more flexible functional form $X_1 = \sum_{i<j} a_{ij} \Theta(\Delta_{ij} - r_{ij})$. The FEP along the optimum coordinate is shown on Fig. 2b. The transition state for the optimum reaction coordinate is not the barrier nearest to the native state

($x = 31$), as suggested by the sub-optimal coordinate, but the one before ($x = 22$). Since this barrier has lower height for the sub-optimal reaction coordinate ($x = 18$), the dynamics over it along the sub-optimal reaction coordinate is sub-diffusive $\alpha \approx 0.25$.

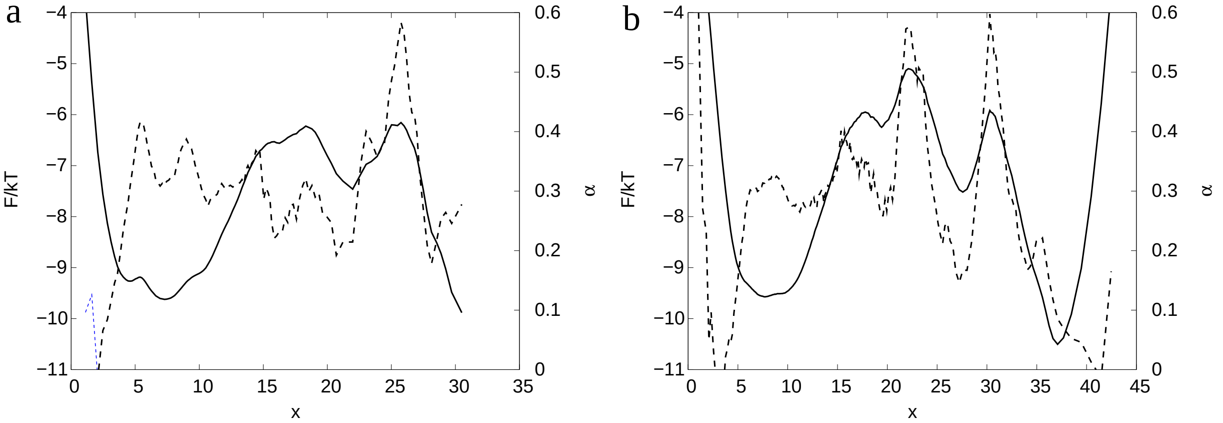

Figure 2. Optimization of X_1 reaction coordinate for all-β protein. F_C (solid line) and $\alpha(x)$ (dashed line) for X_1 as a reaction coordinate; (a) optimized $X_1 = \sum_{ij} \Theta(\mu r_{ij}^0 - r_{ij})$, (b) optimized $X_1 = \sum_{i<j} a_{ij}\Theta(\Delta_{ij} - r_{ij})$. Reaction coordinates are transformed to the natural reaction coordinate.

All-atom structure based model of the lambda repressor protein.

Equilibrium folding of the lambda repressor protein was simulated with an all-atom structure based potential [2]. The all atom model and input files to simulate the protein with the Gromacs package [3] were obtained from the SMOG web site [4]. The all atom Go model was generated with contact map defined with cut-off procedure with default threshold of 4.0 Å; all other parameters were left to default. Langevin dynamics at temperature close to equilibrium (T=110, in Gromacs units) was performed with the times step of 0.0005 (in Gromacs units) for 2.8×10^8 steps; coordinates were saved every 1000 steps.

Fig. 3a shows the FEP along the number of native contacts reaction coordinate, with the distance threshold proportional to the native distance for each native contact $X_1 = \sum_{ij} \Theta(\mu r_{ij}^0 - r_{ij})$; $\mu = 4$ gives the highest barrier at $x = 20$. One can notice three basins: native ($x > 20$), intermediate ($10 < x < 20$) and denatured $x < 10$. We did not perform the detailed comparison between the free energy landscapes of the C_α and the all atom Go models.

The subdiffusion exponent ($\alpha(x) \sim 0.3$) shows that the dynamics, when projected on the numbed of native contacts reaction coordinate is subdiffusive. The optimum reaction coordinate is constructed by optimizing the more flexible functional form $X_1 = \sum_{i<j} a_{ij}\Theta(\Delta_{ij} - r_{ij})$. The FEP along the optimum coordinate is shown on Fig. 3b. The transition state barrier is higher than that for the sub-optimal coordinate (Fig. 3a). The subdiffusion exponent shows that the dynamics around the transition state is diffusive ($\alpha \sim 0.5$).

In fact, it is possible to make dynamics diffusive for the whole coordinate, not just around the transition state. The reaction coordinate is optimized by numerically minimizing the following functional $\int lnZ_C(x)Z_H(x)dx$. If $Z_C(x)$ at different positions can be treated as independent (the reaction coordinate is very flexible), the functional attains minimum when $Z_C(x)$ is minimal for every x; i.e., the profile is highest for every value of the reaction coordinate. This functional optimizes the FEP more uniformly compare to the mean first passage time (Eq. 4), which optimizes the FEP mainly around the transition state. Fig. 4 shows the FEP along thus optimized reaction coordinate, which is uniformly

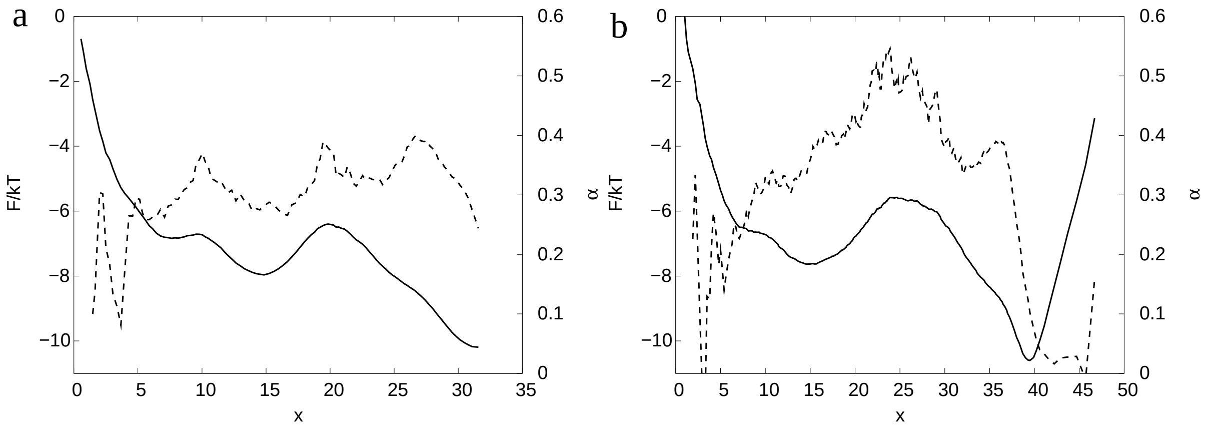

Figure 3. All atom structure based model of λ-repressor. F_C (solid line) and $\alpha(x)$ (dashed line) for X_1 as a reaction coordinate; (a) optimized $X_1 = \sum_{ij} \Theta(\mu r^0_{ij} - r_{ij})$, (b) optimized $X_1 = \sum_{i<j} a_{ij}\Theta(\Delta_{ij} - r_{ij})$. Reaction coordinates are transformed to the natural reaction coordinate.

higher than that along the sub-optimum coordinate 3a. The subdiffusion exponent is around 0.5 for the whole reaction coordinate, indicating diffusive dynamics.

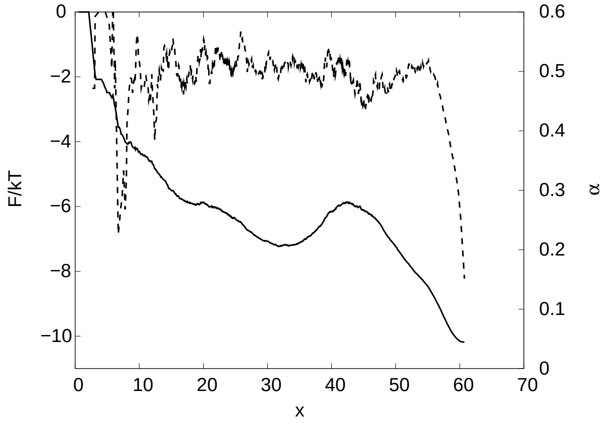

Figure 4. Optimization of the whole reaction coordinate. F_C (solid line) and $\alpha(x)$ (dashed line) for X_1 as a reaction coordinate. Reaction coordinates are transformed to the natural reaction coordinate.

The presented examples show, that the suggested picture that the dynamics is sub-diffusive when projected on a sub-optimal reaction coordinate, while diffusive when projected on the optimum reaction coordinate is robust.

References

1. Brockwell DJ, Paci E, Zinober RC, Beddard GS, Olmsted PD, et al. (2003) Pulling geometry defines the mechanical resistance of a [beta]-sheet protein. Nat Struct Mol Biol 10: 731–737.

2. Whitford PC, Noel JK, Gosavi S, Schug A, Sanbonmatsu KY, et al. (2009) An all-atom structure-based potential for proteins: Bridging minimal models with all-atom empirical forcefields. Proteins: Structure, Function, and Bioinformatics 75: 430–441.

3. Hess B, Kutzner C, van der Spoel D, Lindahl E (2008) GROMACS 4: Algorithms for highly efficient, Load-Balanced, and scalable molecular simulation. Journal of Chemical Theory and Computation 4: 435–447.

4. Noel JK, Whitford PC, Sanbonmatsu KY, Onuchic JN (2010) SMOG@ctbp: simplified deployment of structure-based models in GROMACS. Nucl Acids Res : 1-5.
